# Monoclonal Antibody Targeting of Fibroblast Growth Factor Receptor 1c Ameliorates Obesity and Glucose Intolerance via Central Mechanisms

**DOI:** 10.1371/journal.pone.0112109

**Published:** 2014-11-26

**Authors:** Christopher J. Lelliott, Andrea Ahnmark, Therese Admyre, Ingela Ahlstedt, Lorraine Irving, Feenagh Keyes, Laurel Patterson, Michael B. Mumphrey, Mikael Bjursell, Tracy Gorman, Mohammad Bohlooly-Y, Andrew Buchanan, Paula Harrison, Tristan Vaughan, Hans-Rudolf Berthoud, Daniel Lindén

**Affiliations:** 1 Cardiovascular & Metabolic Disease Innovative Medicines, Dept of Bioscience Diabetes, AstraZeneca, Mölndal, Sweden; 2 Discovery Sciences Transgenics, AstraZeneca, Mölndal, Sweden; 3 Antibody Discovery and Protein Engineering, MedImmune, Cambridge, United Kingdom; 4 Neurobiology of Nutrition Laboratory, Pennington Biomedical Research Center, Baton Rouge, United States of America; 5 AstraZeneca, Discovery Sciences, Mereside, Alderley Park, Macclesfield, Cheshire, United Kingdom; Institut d'Investigacions Biomèdiques August Pi i Sunyer, Spain

## Abstract

We have generated a novel monoclonal antibody targeting human FGFR1c (R1c mAb) that caused profound body weight and body fat loss in diet-induced obese mice due to decreased food intake (with energy expenditure unaltered), in turn improving glucose control. R1c mAb also caused weight loss in leptin-deficient *ob/ob* mice, leptin receptor-mutant *db/db* mice, and in mice lacking either the *melanocortin 4 receptor* or the *melanin-concentrating hormone receptor 1*. In addition, R1c mAb did not change hypothalamic mRNA expression levels of *Agrp*, *Cart*, *Pomc*, *Npy*, *Crh*, *Mch*, or *Orexin*, suggesting that R1c mAb could cause food intake inhibition and body weight loss via other mechanisms in the brain. Interestingly, peripherally administered R1c mAb accumulated in the median eminence, adjacent arcuate nucleus and in the circumventricular organs where it activated the early response gene *c-Fos*. As a plausible mechanism and coinciding with the initiation of food intake suppression, R1c mAb induced hypothalamic expression levels of the cytokines *Monocyte chemoattractant protein 1* and *3* and ERK1/2 and p70 S6 kinase 1 activation.

## Introduction

According to the World Health Organization (WHO), obesity has more than doubled since 1980 and in 2008 at least 1.5 billion adults were overweight and 500 million were obese worldwide, resulting in an increased incidence of type 2 diabetes, cardiovascular disease and premature deaths (www.who.int). Recently, the Fibroblast Growth Factor Receptor 1 (*FGFR1*) SNP rs7012413*T was found to be associated with obesity in four different cohorts [1]. In addition, adipose tissue *FGFR1* mRNA and protein levels were elevated in obese subjects and *Fgfr1* mRNA levels were increased in the hypothalamus of diet-induced obese (DIO) rats [1], showing that *FGFR1* is a novel human obesity candidate gene that may affect metabolism and control of food intake.

The mammalian Fibroblast Growth Factor (FGF) family consists of 22 members and there are 4 FGFRs identified existing in different splice variants with different ligand-binding specificity, reviewed in [2], [3]. Antagonizing FGFR1c with the monoclonal antibody (mAb) IMC-A1 caused weight loss due to reversible hypophagia in animals [4]. Paradoxically, an FGFR1-activating mAb has also been found to cause body weight loss in mice via a combination of both decreased food intake and increased energy expenditure [5].

Here, we describe the identification of a novel fully human FGFR1c targeting mAb (R1c mAb) possessing both antagonistic and agonistic properties that caused in DIO mice profound body weight and body fat loss via reversible hypophagia leading to improved glucose control. Importantly, R1c mAb accumulated and increased neuronal activity in the median eminence, adjacent arcuate nucleus and in other circumventricular organs. As the basis for a plausible mechanism, R1c mAb induced a specific subset of chemokines and activated ERK1/2 and p70 S6 kinase 1in the hypothalamus coinciding with the initial time-course of the food intake suppression.

## Materials and Methods

### Ethics Statement

All animal experiments were approved by the Gothenburg Ethics Committee for Experimental Animals.

### Phage display identification of an anti-FGFR1c monoclonal antibody

Phage display selections were performed according to the methods described in Dobson *et al* using naïve human antibody libraries [6]. Multiple rounds of phage display selection were performed using biotinylated human FGFR1c-extracellular domain (ECD) produced by MedImmune, with deselection using unlabelled human FGFR1b Fc-fusion protein (R&D Systems, Minneapolis, MN). To identify antibodies capable of specific FGFR1c antagonism, crude bacterial peri-plasmic extracts containing scFv antibodies from the selection outputs were prepared [6] and analyzed in an assay designed to measure the binding of FGF2 (produced by MedImmune) to FGFR1c. Full length human FGF2 (UniProt: P09038), fused to a *C*-terminal hexa-histidine tag, was expressed in *E. coli* Rossetta (DE3) pLysS (Merck KGaA, Darmstadt, Germany). Expressed protein was purified by immobilised nickel chromatography followed by size exclusion chromatography. The binding of flag-tagged FGF2 to cryptate labelled FGFR1c-ECD-Fc (R&D Systems) was detected using an XL665 labelled anti-Flag antibody (Cisbio, France) and inhibitors of this interaction were identified. A similar assay to measure inhibition of FGF2 binding to FGFR2c was used as negative screen. FGFR1c specific ScFv were converted to IgG. FGFR1c specific IgG was further profiled in FGF2 induced proliferation using BaF3huFGFR1c cells and a FGF2 induced Ca^2+^ release assay in NIH3T3huFGFR1c cells. The most potent *in vitro* antagonists were selected to test *in vivo*. Fragment antigen-binding (FAb) fragments were generated by papain (Sigma) digestion of R1c mAb IgG followed by MabSelect SuRe (GE Healthcare) purification.

### Receptor binding and specificity assay

Monomeric human FGFR1c (FGFR1βIIIc), FGFR2c (FGFR2βIIIc), FGFR3c, and FGFR4 were produced by MedImmune. The extracellular domains of human FGFRs were fused to a *C*-terminal FLAG epitope tag and a deca-histidine tag and were expressed in human embryonic kidney (HEK) cells using an episomal transient expression system. Purification was achieved by ultra-filtration followed by immobilised nickel chromatography and size exclusion chromatography.

The Octet RED system (ForteBio, UK) was used for real-time, label-free analysis of R1c mAb interaction with FGFR1c. All Octet experiments were run at RT and reagents were prepared in 0.1% BSA, 0.02% Tween20 PBS, pH 7.4 kinetic buffer. Streptavidin sensors were pre-soaked off-line in 200 µl of kinetic buffer and loaded with biotinylated Protein G (Thermo Scientific, UK) prior to loading with 10 µg/ml of R1c mAb or a control mAb. The receptor association phase was measured by incubating the sensor in a titration curve of FGFR1c ECD or kinetic buffer blank for 10 min. The receptor dissociation phase was subsequently measured by incubating all the sensors in kinetic buffer for 10 min. After data processing to subtract reference trace, data analysis was performed using the Data Analysis software package provided with the Octet RED system. Binding to the other FGFRs was assessed by ELISA. Nunc maxisorp plates were coated with 0.5 µg/ml of FGFR overnight, blocked with 3% Marvel-PBS buffer and then washed with PBS. Anti-FGFR or control antibodies, ranging from 0.4 to 100 nM, were added and following incubation plates were washed and detection anti-human Fc-HRP added. The HRP substrate TMB (3, 3', 5, 5'-tetramethylbenzidine) was added and the reaction stopped and read at OD 450 nm using an Envision plate reader.

### FLIPR ligand induced Ca^2+^ Release Assays

To study antibody effects on Ca^2+^ release stimulated by FGFs 1, 2, 4, 5, and 6, NIH3T3 cells overexpressing human FGFR1c were seeded at a density of 7,000 cells per well in 25 µl of DMEM, 10% FBS, 2 µg/ml puromycin and incubated overnight at 37°C/5% CO_2_. Media was then removed and 25 µl of Fluo-4 NW, reconstituted as described by the manufacturer (Invitrogen, Carlsbad, CA) including 2.5 nM probenicid, was added to each well and plates were incubated for 30 min at 37°C/5% CO_2_. All reagents were prepared in 20 mM HEPES, 0.1% BSA, HBSS assay buffer. After equilibrating the cells to RT for 10 min, R1c mAb or control mAb was added to the plates and incubated for 15 min at RT. Final assay concentrations were 0.5 nM FGF1, 0.5 nM FGF2, 4 nM FGF4, 40 nM FGF5, or 4 nM FGF6. For FGF4 and FGF6 assays, heparin was added in the pre-incubation step. FGF1, FGF2 and FGF5 assays did not require heparin. FGF agonist was then added to the plates and Ca^2+^ transients were recorded immediately on a FLIPR Tetra (Molecular Devices, UK). Fluorescence was excited at 470_495 nm and emission was measured at 515_575 nm. Data was analyzed by taking maximum peak height minus background and calculating % of maximum response.

### Transfection of BaF3 Cells and Proliferation Assays

BaF3 cells (murine interleukin-3 dependent pro-B cell line) overexpressing human FGFR1c were kindly provided by Prof. David Ornitz (Washington University School of Medicine, St Louis, Missouri, USA). BaF3huFGFR1c cells were maintained in RPMI 1640 (Invitrogen) medium supplemented with 8.8% Newborn bovine serum, 0.04% mouse IL3, 0.09% 2ME, 1.8% Glutamax-I and 1% G418. BaF3huFGFR1c cells were transfected with α-Klotho or β-Klotho in pEF-DEST51 expression vector (Invitrogen) by nucleofection using the Amaxa Nucleofector Kit V (Lonza). Transfectants expressing α-Klotho or β-Klotho were selected and maintained by incubation in the presence of 4 µg/ml blasticidin.

Proliferation of cells in the presence of FGF ligands was measured using a Luminescent Cell Viability Assay (Promega, USA). Briefly, the transfectants were washed to remove IL3 and plated to a density of 8,000 cells per well in 384-well plates. BaF3huFGFR1c cells were used for the FGF2 assay, BaF3huFGFR1c/β-klotho cells were used for the FGF19 and FGF21 assays, and BaF3huFGFR1c/α-klotho cells were used for the FGF23 assay. The proliferation assay media was RPMI 1640 (Invitrogen, Carlsbad, CA) medium supplemented with 8.8% Newborn bovine serum, 0.09% 2ME, 1.8% Glutamax-I, 1% G418 and 10 ug/ml heparin. The final FGF concentrations were 0.02 nM FGF2, 50 nM FGF19, 50 nM FGF21, or 0.02 nM FGF23. The cells were stimulated with FGFs with or without R1c mAb or control mAb and incubated at 37°C for 72 h. Prior to addition of CellTiterGlo reagent (Promega, USA), the cells were equilibrated to room temperature for 30 min. Luminescence was read on an Envision Multilabel reader (Perkin Elmer, UK).

### Animals, diets, food intake analysis and body composition

DIO C57BL/6J mice (Harlan, the Netherlands) had free access to a high-fat diet (HFD) containing (energy percentage) 60% fat, 20% carbohydrates, and 20% protein, with a total energy content of 5.2 Kcal/g (D12492; Research Diets, New Brunswick, NJ) for 6–12 weeks before treatment starts. A pair-feeding experiment was performed over four days in which one group of DIO mice dosed with a single injection of R1c mAb were fed *ad libitum* and two groups of DIO mice receiving a single injection of control mAb were fed either *ad libitum* or pair-fed twice daily to match the food intake of R1c mAb treated DIO mice fed *ad libitum*. Leptin-deficient *ob/ob* mice, leptin receptor-mutant *db/db* mice (Harlan), and melanocortin receptor 4 (*Mc4r*)-deficient mice [7] were kept on regular chow diet containing (energy percentage) 12% fat, 62% carbohydrates, and 26% protein, with a total energy content of 3 Kcal/g (R3; Lactamin, Kimstad, Sweden) and melanin-concentrating hormone receptor (*Mchr*)-deficient mice [8] were kept on HFD (D12492; Research Diets). Cumulative food intake was analyzed in single housed mice in an automated food intake analysis system (AstraZeneca, Mölndal, Sweden). The mice were adapted to the system for at least 5 d before treatment start and statistical analyses were performed on bi-hourly food intake during the first 12 h post treatment start.

Body composition analysis was performed on isoflurane (Forene, Abbot Scandinavia AB, Sweden) anesthetized mice with dual-energy X-ray absorptiometry (DEXA, PIXImus Lunar, GE Medical Systems, Madison, WI). At termination (9–11 a.m.), plasma was isolated from isoflurane anesthetized mice and organs were collected, weighed and snap frozen in liquid N_2_ and stored at −80°C.

### Plasma biochemistry

Plasma total adiponectin levels were measured using a radioimmunoassay from Linco Research (St. Charles, MO) and plasma leptin with an ELISA from Chrystal Chem. Inc. (Downers Grove, IL). Plasma cholesterol levels were measured with a kit from Roche Diagnostics (Mannheim, Germany), alanine aminotransferase (ALT) with a kit from ABX Diagnostics (Montpellier, France), β-hydroxybutyrate with a kit from Randox Laboratories (Antrim, UK) and non-esterified fatty acids (NEFAs) with a kit from WAKO Chemicals (Neuss, Germany).

### Oral glucose tolerance test

Oral Glucose Tolerance Tests (OGTTs) were performed 1 p.m. after a 4 h fast by oral administration of 2 g glucose per kg body weight. Blood (12 µl) was sampled from the tail vein at 0, 15, 30, 60 and 120 min for determination of glucose (2 µl, Accu-Chek, Roche Diagnostics, Mannheim, Germany) and insulin (2×5 µl, Ultra-sensitive mouse insulin ELISA kit, Crystal Chem. Inc., Downers Grove, IL) levels.

### Indirect calorimetry and spontaneous locomotor activity

Oxygen consumption (vO_2_) and carbon dioxide production (vCO_2_) were measured using a CLAMS open circuit calorimetry system (Oxymax, Columbus Instruments, Columbus, OH). Energy expenditure (Kcal/h) was calculated: (3.815+1.232RER)×vO_2_, where RER is the respiratory exchange ratio [volume of CO_2_ produced per volume of O_2_ consumed (both ml/kg/min)] and vO_2_ is the volume of O_2_ consumed per h per kg mass of the animal. The value of energy expenditure was correlated to individual body weights. Horizontal spontaneous locomotor activity was measured continuously in the CLAMS system.

### Assessment of general behavior

An elevated zero maze system (Accuscan Instruments Inc., Columbus, OH) was used to assess anxiety related behavior as previously described [9]. Mice were acclimatized once to the system 3–4 h after dosing and analyzed 24 h later. Memory and learning ability was assessed by a passive avoidance approach in the Shuttle box system (Accuscan Instruments Inc., Columbus, OH) as described [9]. Assessments were performed 50 h after dosing and then repeated 75 h after dosing. To assess signs of depression, a forced swim test approach was used in water filled plexiglas cylinder (AstraZeneca R&D, Mölndal, Sweden). Active swim time was assessed during the last 4 minutes of the test as previously described [9]. Assessment of depression was performed 50 h post dosing.

### Pancreas α- and β-cell mass

Sample preparation and immunohistochemistry were performed according to Sun *et al* [10]. Isolated pancreas were fixed in 10% buffered formalin and embedded in paraffin wax within 24 h of removal. Head-to-tail sections (4 µm lengthwise) were cut and incubated overnight at 37°C on superfrost slides. Slides were submerged sequentially in xylene followed by decreasing concentrations of industrial methylated spirits for removal of paraffin wax. Slides were blocked in 5% (vol./vol.) goat serum in Tris buffered saline with 0.05% (vol./vol.) Tween (TBST) for 20 min at room temperature then incubated sequentially with primary (1∶200 mouse anti-glucagon ab10988, Abcam, Cambridge, UK) and secondary glucagon antibodies (1∶200 rabbit anti-mouse IgG antibody∶TRITC conjugate; T-2402, Sigma), for 60 minutes followed by a further block in 5% goat serum in TBST prior to sequential incubation in primary (1∶50 Guinea pig anti-insulin, Dako A0564) and secondary insulin antibodies (1∶400 goat anti guinea pig AlexaFluor 488, A11073, Invitrogen). Hoeschst (2 µM) was included in the final incubation as a fluorescent nuclear stain and sections were mounted using FluorSave mounting Reagent (Merck 345789). Six longitudinal sections, at 40 micron intervals were imaged per pancreas, with the SpGreen and SpOrange filters respectively on a Zeiss Axio Imager M1 (Carl Zeiss, Jena, Germany) operated through the Metafer software (MetaSystems, Waltham, MA, USA). A custom designed Metafer software classifier enabled mapping of all pancreatic tissue. Definiens image analysis software (Munich, Germany) quantified all fluorescent images. The results are expressed as a percentage of total pancreatic area.

### RNA preparation and quantitative real-time PCR

Total RNA was extracted using Qiazol and disruption of the sample in a Mixer Mill 300 (Qiagen, Valencia, CA). RNA clean-up and DNase digestion were performed using RNeasy Mini Kit (Qiagen). RNA content and quality was assessed using Nanodrop (ThermoScientific, Wilmington, DE). First strand cDNA was synthesized using the High Capacity cDNA Archive Kit (Applied Biosystems, Foster City, CA). Real-time PCR analysis was performed with an ABI Prism 7900 Sequence Detection System (Applied Biosystems) using 5′-FAM and 3′-TAMRA labeled fluorogenic oligonucleotide probes or primers designed for use for SYBR-Green detection. Primers/probes were purchased from Sigma-Genosys (Haverhill, UK). The relative expression levels were calculated by using the standard curve method in order to define an arbitrary expression level. This data was then normalized against mouse acidic ribosomal phosphoprotein P0 (*m36B4*) expression for each sample. Sequences for primers and probes are presented in Table 1.

**Table 1:** Primer and Probe sequences for quantitative RT-PCR.

Gene	Forward (5′-3′)	Reverse (5′-3′)	Probe (5′-3′)
***Agrp***	CGGAGGTGCTAGATCCACAGA	AGGACTCGTGCAGCCTTACAC	CGAGTCTCGTTCTCCGCGTCGC
***Cart***	CTGCAATTCTTTCCTCTTGAAGTG	GGGAATATGGGAACCGAAGGT	SYBR
***Ccr2***	AGAAGTTCCGAAGGTATCTCTCCAT	CCCTATAGAAAACTGGGCACTGTT	TTTTTCAGAAAGCACATTGCTAAACGTCTCTGC
***Ccr5***	CTTCATGTTAGATTTGTACAGCTCTCCTA	CATCCTGCAAGAGCCAGAGTCT	CCAGAGGAGGTGAGACATCCGTTC
***Cd11c***	CCACTGTCTGCCTTCATATTCATG	CATGGTCTAGAGCCAGGTCAAAG	CCAAGACCCAACTAGGTGACCTCCGAAGTA
***Crh***	GGAGCCGCCCATCTCTCT	CGGGCCATTTCCAAGACTT	SYBR
***Cx3cl1***	CGCGTTCTTCCATTTGTGTACTC	GCACATGATTTCGCATTTCG	CTGCCGGGTCAGCACCTCGG
***Cxcl12***	CTGAAAATCCTCAACACTCCAAACT	GCACACTTGTCTGTTGTTGTTCTTC	TGCCCTTCAGATTGTTGCACGGC
***Cxcl5***	GCTGGCATTTCTGTTGCTGTT	GCTCCGTTGCGGCTATGA	CGCTGCCGCAGCATCTAGCTGAAG
***Il-1β***	AATCTATACCTGTCCTGTGTAATGAAAGAC	TGGGTATTGCTTGGGATCCA	CACACCCACCCTGCAGCTGGAGA
***Ifnγ***	CAGCAACAGCAAGGCGAAA	CTGGACCTGTGGGTTGTTGAC	AGGATGCATTCATGAGTATTGCCAAGTTTGA
***Lif***	CAACCTCATGAACCAGATCAA	AAAGATGGGAAGTCTGTCATGTT	AGAGAGCATTGGCGCTGCCA
***Mch***	TTCAGAAGGAAGATACTGCAGAAAGA	GCTCTCGTCGTTTTTGTATTGTT	SYBR
***Mcp1/Ccl2***	GCTGGAGAGCTACAAGAGGATCA	TCTCTCTTGAGCTTGGTGACAAAA	AGCAGCAGGTGTCCCAAAGAAGCTGTAG
***Mcp2/Ccl8***	AGCTACGAGAGAATCAACAATATCCA	CAGAGAGACATACCCTGCTTGGT	TGCCCCATGGAAGCTGTGGTTTTC
***Mcp3/Ccl7***	CAAGAGCTACAGAAGGATCACCAGTA	GACTTCCATGCCCTTCTTTGTC	TCGGTGTCCCTGGGAAGCTGTTATCTT
***Npy***	TACTCCGCTCTGCGACACTACA	AATCAGTGTCTCAGGGCTGGAT	SYBR
***Orexin***	CTGCCGTCTCTACGAACTGTTG	CGCTTTCCCAGAGTCAGGATA	SYBR
***Pgc-1α***	GTAGGCCCAGGTACGACAGC	GCTCTTTGCGGTATTCATCCC	ATGAAGCCTATGAGCACGAAAGGCTCAA
***Pgc-1β***	CTCCAGGCAGGTTCAACCC	GGGCCAGAAGTTCCCTTAGG	CCCCCCAAAGCCTTCTGGACTGAGT
***Pomc***	TGCTTCAGACCTCCATAGATGTGT	GGATGCAAGCCAGCAGGTT	SYBR
***Ptgs1***	TGGCCACATTTATGGAGATAAT	GGACACCTGGTGGGTAGC	CGTGGAACAGGCGTCCGTGT
***Ptgs2***	TCCAACCTCTCCTACTACACCA	GGGTCAGGGATGAACTCTCT	TGCTACGGGAGGAAGGGCCC
***Socs3***	CCGCGGGCACCTTTC	TTGACGCTCAACGTGAAGAAGT	CCGCGACAGCTCGGACCAGC
***Tlr4***	TCTGATCATGGCACTGTTCTTCTC	TCTGATCCATGCATTGGTAGGT	CCAGGAAGCTTGAATCCCTGCATAGAGGTAGT
***Tnf-α***	ATGGCCCAGACCCTCACA	TTGCTACGACGTGGGCTACA	TCAGATCATCTTCTCAAAATTCGAGTGACAAGC
***Ucp1***	CTGGGCTTAACGGGTCCTG	CTGGGCTAGGTAGTGCCAGTG	AGCTCAAAGGTGAGCGTCCCTTCCTC

### Tissue and cell protein extraction and Western blot

Hypothalamus or white adipose tissue protein extracts were generated by homogenization in ice cold T-PER protein extraction reagent (Pierce, Rockford, IL) containing a protease inhibitor cocktail (Complete Mini and phosphoSTOP, Roche Diagnostics), with a single-use pestle in a 1.5 ml Eppendorf Tube (VWR, Radnor, PA). Homogenates were centrifuged (4°C, 20,000×g for 15 min) and the protein content of the lysate quantitated using BCA Assay Reagent (Pierce). Mouse embryonic hypothalamic N46 cells were from Cellutions Biosystems (Burlington, Canada) and cultured in Dubecco's Modified Eagle Medium (DMEM) (Sigma) supplemented with 10% FBS (Hyclone, Erembodegem, Belgium). Confluent cells were serum starved in medium containing 5 µg/ml heparin for 16 h before experiment. Mouse 3T3-L1 fibroblasts (American Type Culture Collection, Middlesex, UK) were maintained in DMEM supplemented with 10% calf serum and 1% PEST (Invitrogen). Confluent 3T3-L1 cells were differentiated in DMEM, 10% FBS (PAA gold), 1% PEST, 1 µM Dexamethasone (Sigma), 0.5 mM IBMX (Sigma) and 0.17 µM insulin (Novo Nordisk, Denmark). Cells were serum starved in OptiMEM I (Invitrogen) containing 0.5% essentially fatty acid free BSA (Sigma) 16 h before experiment. Cells were incubated with or without 50 µg/ml antibodies for 1 h with or without 10 ng/ml FGF2 (MedImmune) for 10 min (PBS was used as control). Cultured cells were lysed in lysis buffer (50 mM Tris-HCl, pH 7.5, 1 mM EGTA, 1 mM EDTA, 50 mM sodiumfluoride, 5 mM pyrophosphate, 0.27 M sucrose, 1% (v/v) Triton-X 100 and 1 mM sodium orthovanadate) with a protease inhibitor cocktail and phosphoSTOP as above. Primary antibodies for Western blot analyses were from Abcam [pPDK #52893], Sigma [α-Tubulin #T6074] or from Cell Signaling Technology (Beverley, MA), pFGFR (Tyr653/654 #3471), pPLCγ1 (Tyr783 #2821), pFRS2α (Tyr436 #3861), pAkt (Ser473 #4058L), pERK1/2 (Thr202/Tyr204 # 4377), pStat3 (Tyr705 #9131S), pp70S6K1 (Thr389 #9205), pMEK (#9154S), pJak2 (Tyr1007/1008 #3776S), pJNK (#4668P), and pp38 MAPK (T180/Y182 #4511P). HRP-conjugated polyclonal goat anti-Rabbit P0448 (DAKO) was used as secondary antibody except for the anti α-Tubulin antibody where an HRP conjugated goat anti-mouse P0447 (DAKO) was used. Enhanced chemiluminescence reagents (Pierce) were used for detection.

### Immunohistochemical detection of c-Fos, human IgG and P-ERK in the brain

Isoflurane anesthetized mice were transcardially perfused with heparinized saline, followed by ice-cold 4% phosphate-buffered (pH 7.4) paraformaldehyde. Brains were held at −20°C in cryoprotectant (50% PBS, 30% ethylene glycol, 20% glycerol), removed from the skull and cut into three blocks (forebrain, midbrain, and hindbrain). After multiple washes with PBS, the blocks were soaked in a sucrose solution (18% sucrose, 0.05% sodium azide in 1.0 M PBS) for cryoprotection. Blocks were then frozen and cut into 30 µm coronal sections in a cryostat microtome. Sections were separated into 5 series. For immediate processing, sections were held in PBS at 4°C, and for long term storage, in a cryoprotectant solution at −20°C. With appropriate washes in PBS between incubations, free-floating sections were pretreated with 0.5% sodium borohydride in PBS to minimize aldehyde cross-linking of the fixative. Sections were then treated with a hydrogen peroxide solution (1.5% H_2_O_2_, 20% methanol, 0.25% Triton X-100 in PBS) to minimize endogenous peroxidase activity. Sections were finally placed in a blocking solution of 0.5% normal donkey serum in PBS containing 0.5% Triton X-100 (PBST). The sections were incubated in primary antibody (rabbit anti-c-Fos, 1∶15 000; EMD Biosciences, La Jolla, CA, USA) in primary diluent (PBST containing 0.1% gelatin and 0.05% sodium azide) for 24 hours at room temperature, or up to 48 hours at 4°C. They were then incubated in secondary antibody (biotinylated donkey anti-rabbit, 1∶500; Jackson Immuno Research, West Grove, PA, USA) diluted in PBST for 2.5 hours at room temperature. The sections were then treated with an avidin-biotin complex kit (VECTASTAIN Elite ABC Kit, Vector Laboratories, Burlingame, CA, USA) for 1 hour at room temperature. Finally, the sections were visualized in a DAB solution (0.5% 3,3′-diaminobenzadine tetrahydrochloride, 1% cobalt chloride and nickel chloride in stable hydrogen peroxide substrate buffer, Thermo Scientific, Rockford, IL, USA) for 5 to 10 minutes at room temperature. For double labeling, sections received a second incubation in the donkey serum blocking solution. Sections were then incubated in primary antibody (rabbit anti-human-IgG, 1∶1000, AbD Serotec, Kidlington, UK; rabbit anti-pERK, 1∶1000, Cell Signaling Technology, Danvers, MA, USA) in primary diluent for 24 hours at room temperature, or up to 48 hours at 4°C. They were then incubated in secondary antibody (CY3 donkey anti-rabbit, 1∶500, Jackson Immuno Research, West Grove, PA, USA) for 2.5 hours at room temperature. Sections were washed in PBS after every treatment step except between blocking with donkey serum and incubation with primary antibody. All incubations were carried out with gentle agitation from an orbital shaker. Sections were mounted onto Superfrost glass slides using Prolong Gold Antifade Reagent with DAPI (Invitrogen, Grand Island, NY, USA) as the mounting medium. Sections were viewed using a Zeiss Axioplan fluorescence microscope (Carl Zeiss Microscopy, Thornwood, NY, USA). Counts were performed visually.

### Statistics

The animals were randomized to experimental groups based on body weights before treatment start. Differences between two groups were examined for statistical significance using Mann-Whitney *U*-test. For multiple groups, Kruskal-Wallis ANOVA was used followed by Mann-Whitney *U*-test. CLAMS *P*-values were calculated using the MIXED procedure in SPSS. Values are presented as means ± SEM. *P*<0.05 was considered significant.

## Results

### Identification and *in vitro* characterization of the anti-FGFR1c antibody

**Figure 1:**
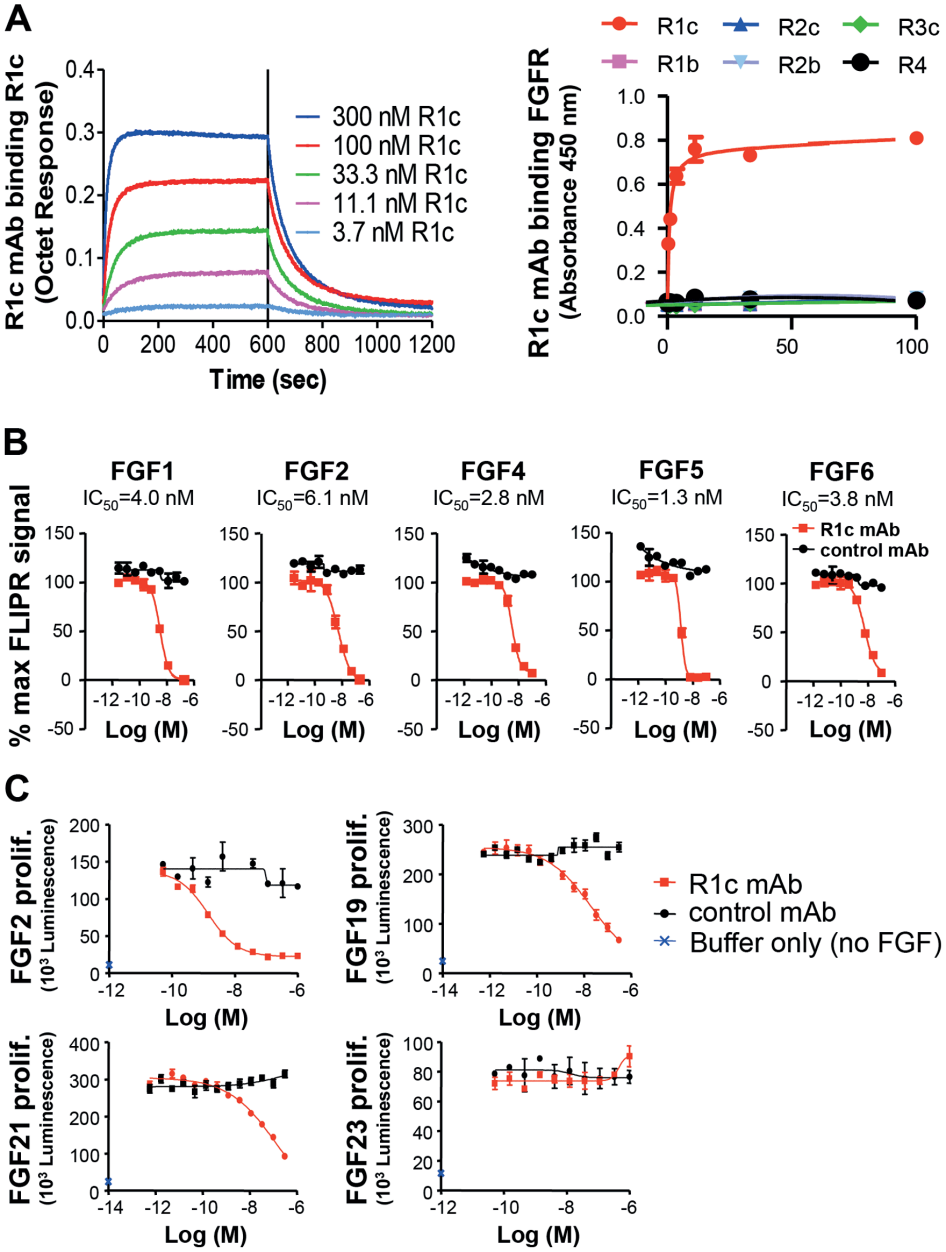
Characterization of anti-FGFR1c (R1c) mAb. (A) Octet association and dissociation kinetic profile for the interaction between R1c mAb and varying concentrations of human FGFR1c. R1c mAb binding to human FGFR1c (R1c), FGFR1b (R1b), FGFR2c (R2c), FGFR2b (R2b), FGFR3c (R3c), or FGFR4 (R4) in an ELISA. (B) R1c mAb inhibition of FGF1, 2, 4, 5, and 6 binding to NIH3T3 cells overexpressing human FGFR1c measured by Ca^2+^ release in a FLIPR assay. (C) R1c mAb inhibition of FGF2, FGF19, FGF21, and FGF23 induced BaF3huFGFR1c cell proliferation. β-klotho was overexpressed in the FGF19 and FGF21 assays and α-klotho was overexpressed in the FGF23 assay. *n* = 3 wells per treatment. Values are means ± SEM.

A monoclonal antibody directed against human FGFR1c (R1c mAb) was identified by scFv phage display selection. On conversion to the IgG form, the R1c mAb bound human and mouse FGFR1c and did not bind to the other FGF receptors FGFR1b, FGFR2b and c, FGFR3c, or FGFR4 (Fig. 1A, data for mouse not shown). R1c mAb inhibited FGF1, FGF2, FGF4, FGF5 and FGF6 induced Ca^2+^ release in NIH3T3 cells overexpressing human FGFR1c (Fig. 1B). R1c mAb also inhibited FGF2, FGF19 and FGF21 induced proliferation of BaF3huFGFR1c cells transfected with β-Klotho (FGF19 and FGF21) but did not affect FGF23 induced proliferation of BaF3huFGFR1c cells transfected with α-Klotho (Fig. 1C). Thus, we have generated an FGFR1c-specific monoclonal antibody which blocks ligand-induced FGFR1c activation.

**Figure 2:**
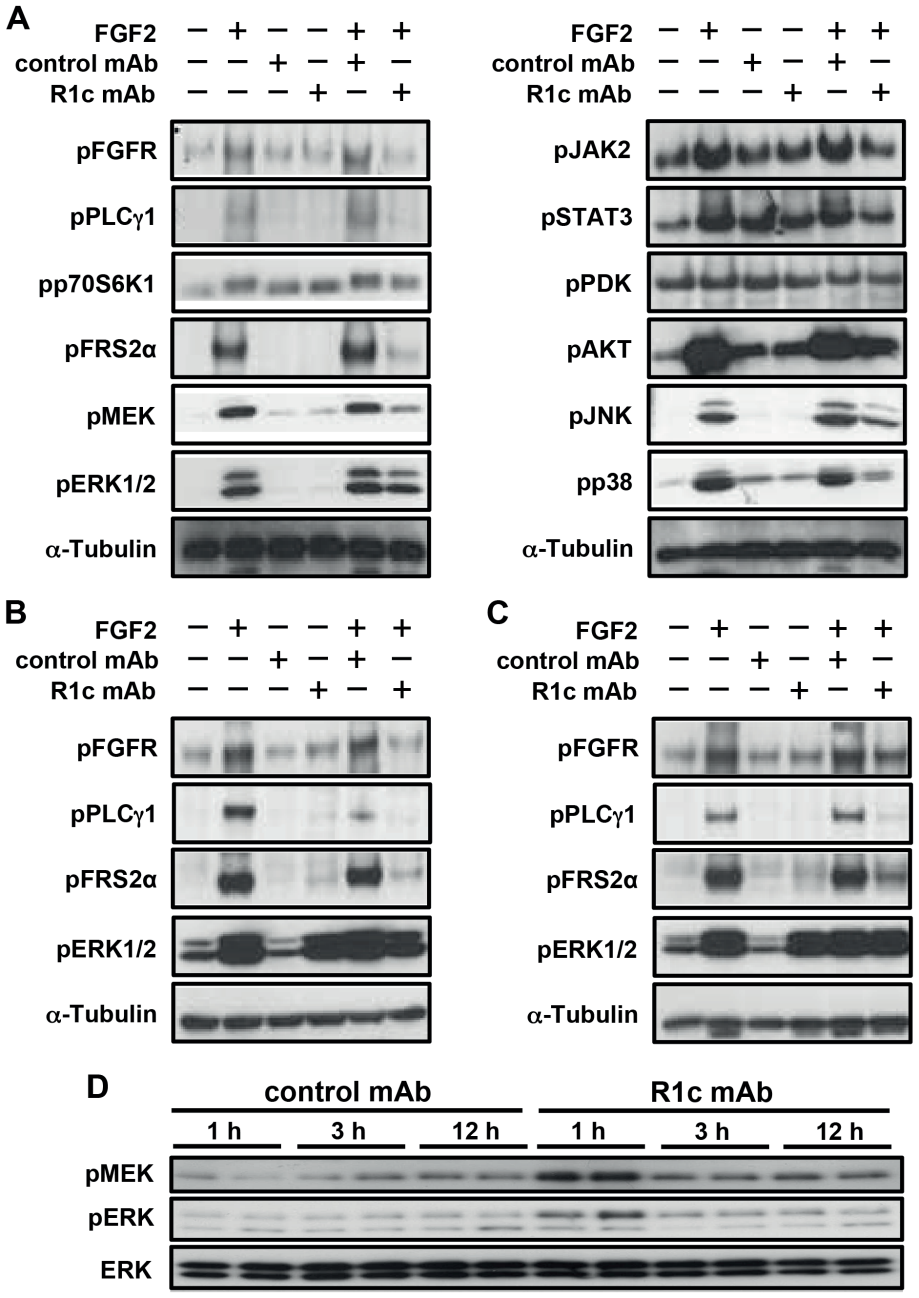
FGFR1c mAb effects on FGFR and intracellular downstream signalling. Representative Western blot analyses on FGFR1c (R1c) mAb (50 µg/ml), control mAb (50 µg/ml) and FGF2 (10 ng/ml) treated (A) mouse embryonic hypothalamic N46 cells, (B) undifferentiated 3T3-L1 cells and (C) differentiated 3T3-L1 cells (D). White adipose tissue Western blot analyses after treatment of female diet-induced obese (DIO) mice with a single injection of either R1c mAb or control mAb (10 mg/kg i.v.). The mice were fasted during the treatment period.

R1c mAb was able to inhibit the FGF2 induced phosphorylation of FGFR and major downstream signaling components including PLCγ1, p70S6K1, FRS2α, MEK, ERK1/2, JAK2, STAT3, AKT, JNK, and p38 (Fig. 2A). Furthermore, R1c mAb inhibited FGF2-induced phosphorylation of FGFR, PLCγ1, FRS2α and ERK1/2 in both undifferentiated and differentiated 3T3-L1 cells. Interestingly, R1c mAb also induced phosphorylation of ERK1/2 in both undifferentiated and differentiated 3T3-L1 cells (Fig. 2B–C). In addition, R1c mAb treatment induced acute phosphorylation of MEK and ERK1/2 in white adipose tissue (WAT) (Fig. 1D), indicating that R1c mAb has dual antagonist/agonist properties.

### R1c mAb reverses obesity and glucose intolerance via decreased food intake

**Figure 3:**
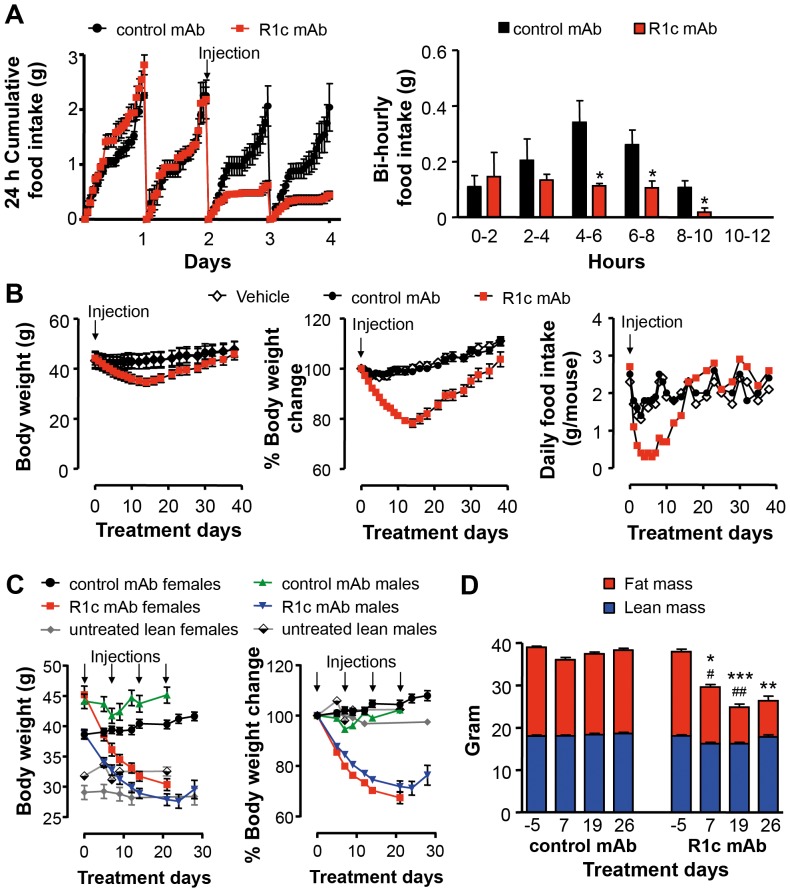
FGFR1c mAb effects on food intake, body weight and body composition. (A) 24 h cumulative food intake and Bi-hourly food intake during the first 12 h following a single injection of either R1c mAb or control mAb in female DIO mice (*n* = 4, 10 mg/kg i.p. indicated with an arrow). *P<0.05 *vs*. control mAb, Mann Whitney *U*-test. (B) Body weight (*n* = 4), percent body weight change (*n* = 4) and food intake (*n* = 1, group housed animals) following a single injection of either vehicle, R1c mAb or control mAb in female DIO mice (10 mg/kg s.c. indicated with an arrow). (C) Body weight and body weight change following repeated injections of either R1c mAb or control mAb in female or male DIO mice (*n* = 7–8, 10 mg/kg i.p. once weekly indicated with arrows) compared with untreated lean mice on chow diet (*n* = 8–10). (D) Quantitative assessment of body fat mass and body lean mass by dual-energy X-ray absorptiometry in female DIO mice (*n* = 7, 10 mg/kg i.p. day 0, 7, 14 and 21). *P<0.05, **P<0.01, and ***P<0.001 R1c mAb fat mass *vs*. control mAb fat mass and ^#^ *P*<0.05, and ^##^ *P*<0.01 R1c mAb lean mass *vs*. control mAb lean mass at corresponding treatment day, Mann Whitney *U*-test. Values are means ± SEM.

Dosing DIO mice with a single i.p. injection of R1c mAb resulted in a significant suppression of food intake commencing 4–6 h post-dose (Fig. 3A). R1c mAb treatment decreased the average number of feeding bouts and the amount of food consumed during each feeding bout 24 h post-treatment while the duration of each bout was not affected up to 36 h post-treatment (data not shown). Thus, although R1c mAb treatment markedly decreased total food intake, it did not disrupt normal meal patterns. Using i.v. injection, food intake inhibition commenced 2–4 h post-dosing, whereas using s.c. delivery, the onset of food intake inhibition was delayed for more than 12 h (data not shown).

A single injection of R1c mAb (T_1/2_ at 10 mg/kg s.c. = 8 d) in DIO mice reduced food intake below vehicle and control mAb levels for 14 days resulting in transient body weight loss (Fig. 3B). A single injection of R1c mAb also reduced body weights in lean chow fed mice (R1c mAb to 91.5±1.4% *vs*. control mAb 99.8±0.7% (means ± SEM) of initial start weight (∼20 g) 2.5 d after the injection, *P<0.01, Mann-Whitney *U*-test, *n* = 6 per group, mAb 10 mg/kg i.v.). Repeated R1c mAb dosing lead to sustained body weight loss in both male and female DIO mice reaching levels of age-matched lean mice (Fig. 3C) associated with reduced body fat mass (Fig. 3D).

R1c mAb treatment did not affect active time, latency to enter open compartments nor the time spent in the closed or open compartments in a zero maze assessment (data not shown). Furthermore, R1c mAb treated mice did not display signs of impaired memory learning ability in a passive avoidance test or any signs of depression in a forced swim test (data not shown), indicating that R1c mAb did not induce general behavior changes in mice.

**Figure 4:**
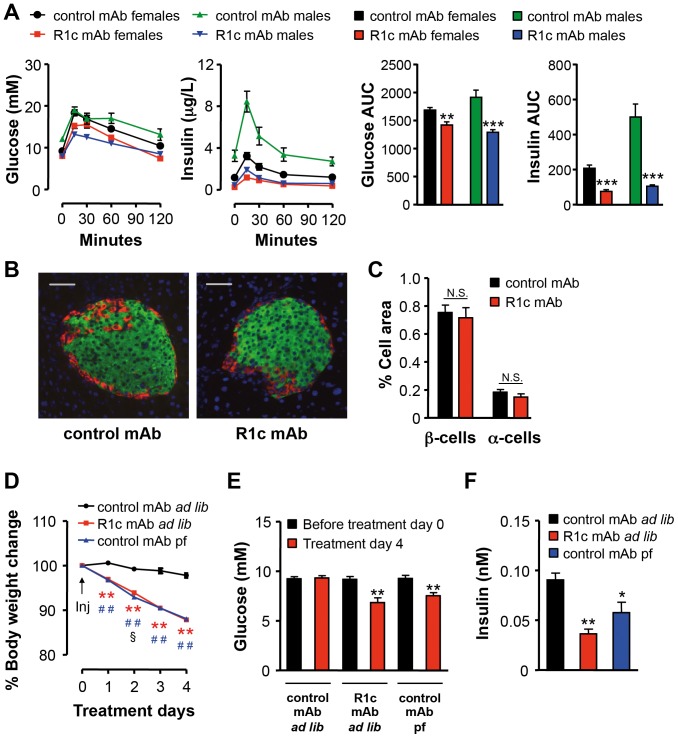
FGFR1c mAb effects on glucose tolerance and pancreatic islet cell mass. (A) Oral glucose tolerance test (OGTT) 15 d after repeated injections of either R1c mAb or control mAb in female or male DIO mice (*n* = 7–8, three i.p. injections 10 mg/kg on day 0, 7 and 14). Glucose and insulin area under the curve (AUC) were calculated. **P<0.01 and ***P<0.001 *vs.* respective control mAb, Mann Whitney *U*-test. (B) Representative pancreatic islet staining of insulin (green), glucagon (red), and nucleuses (blue), and (C) β and α cell quantification after repeated injections with either R1c mAb or control mAb in female DIO mice after 28 d treatment (*n* = 7, 10 mg/kg i.p. day 0, 7, 14 and 21). N.S.  =  Non Significant. (D) Percent body weight change after a single injection of R1c mAb in female DIO mice fed *ad libitum* (*ad lib*) or control mAb treated female DIO mice fed *ad lib* or pair-fed (pf) twice daily to match the food intake in R1c mAb treated female DIO mice fed *ad lib* (*n* = 6, 3 mg/kg s.c., indicated with an arrow). **P<0.01 R1c mAb *ad lib vs.* control mAb *ad lib*, ^##^ *P*<0.01 control mAb pf *vs.* control mAb *ad lib*, and ^§^ *P*<0.05 R1c mAb *ad lib vs.* control mAb pf, Mann Whitney *U*-test. (E) Effects on 3 h fasting glucose levels before treatment start day 0 and after 4 d treatment. **P<0.01 *vs.* Treatment day 0 within respective treatment group, Mann Whitney *U*-test. (F) Effects on 3 h fasting insulin levels after 4 d treatment. *P<0.05, **P<0.01 *vs.* Control mAb fed *ad lib*, Mann Whitney *U*-test. Values are means ± SEM.

R1c mAb treatment improved oral glucose tolerance in both female and male DIO mice (Fig. 4A) and pancreas histology revealed normal α- and β-cell distribution and islet structure (Fig. 4B–C). R1c mAb treatment decreased the WAT and liver weights and plasma ALT and leptin levels in DIO mice compared with control mAb treatment while there was no effect on plasma levels of triglycerides, NEFA or total adiponectin (Table 2). Interestingly, in a pair-feeding experiment, pair-fed control mAb treated DIO mice lost the same amount of body weight and reduced fasting glucose and insulin levels to similar extent as R1c mAb treated DIO mice fed *ad libitum* (Fig. 4D–F). Thus, the R1c mAb induced improvement in glucose control was most likely due to the reduction in food intake and body weight loss.

**Table 2:** FGFR1c (R1c) mAb effects on organ weights and plasma biochemistry.

	Liver	WAT	Triglycerides	NEFA	β-hydroxybutyrate	ALT	Leptin	Adiponectin
	(g)	(g)	(mM)	(mM)	(uM)	(ukat/l)	(ng/ml)	(ug/ml)
**Females**								
**Control mAb**	1.13±0.02	3.14±0.13	0.28±0.01	0.30±0.01	360.1±33.0	1.15±0.13	28.0±2.4	0.87±0.05
**R1c mAb**	1.09±0.06	1.24±0.23^***^	0.29±0.03	0.28±0.01	353.8±21.9	0.77±0.05*	9.8±3.5	0.82±0.07
**Males**								
**Control mAb**	1.71±0.14	2.04±0.15	0.50±0.06	0.46±0.02	299.1±40.1	1.35±0.15	22.1±3.0	0.49±0.03
**R1c mAb**	1.12±0.04^**^	0.94±0.08^***^	0.47±0.03	0.44±0.01	297.7±46.4	0.63±0.04***	3.7±1.3	0.43±0.02

Female or male DIO mice were treated with either R1c mAb or control mAb (*n* = 7–8, 10 mg/kg i.p. once weekly for 3 w, males or 4 w, females). *P<0.05, **P<0.01, ***P<0.001 *vs*. control mAb, Mann Whitney *U*-test. Values are means ± SEM.

**Figure 5:**
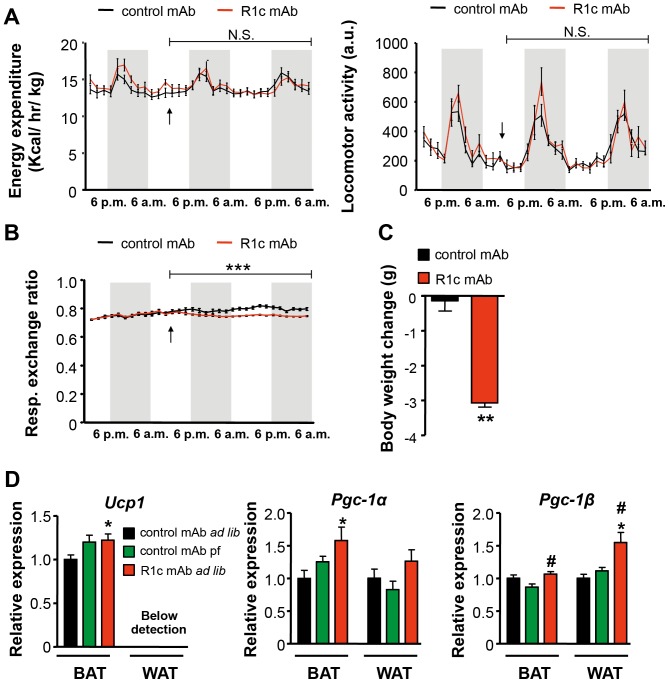
FGFR1c mAb effects on energy expenditure and spontaneous locomotor activity. (A) Energy expenditure, spontaneous locomotor activity and (B) respiratory exchange ratio measured with a CLAMS open circuit indirect calorimetry system following a single injection with either R1c mAb or control mAb in female DIO mice (*n* = 7, 10 mg/kg i.p. indicated with an arrow). ***P<0.001 *vs.* Control mAb, linear mixed model, N.S.  =  Non Significant. (C) Body weight change during the treatment period (42 h). **P<0.01 *vs.* control mAb, Mann Whitney *U*-test. (D) mRNA expression in brown adipose tissue (BAT) and ovarian white adipose tissue (WAT) 4 d after a single injection of R1c mAb in female DIO mice fed *ad libitum* (*ad lib*) or control mAb treated female DIO mice fed *ad lib* or pair-fed (pf) twice daily to match the food intake in R1c mAb treated female DIO mice fed *ad lib* (*n* = 6, 3 mg/kg s.c.). *P<0.05 *vs*. control mAb *ad lib* group; ^#^ *P*<0.05 *vs*. control mAb pf group, Mann Whitney *U*-test. Values are means ± SEM.

There was no significant difference between R1c mAb and control mAb treated female DIO mice on total energy expenditure, spontaneous locomotor activity (Fig. 5A), or core body temperature (data not shown) while RER was decreased following R1c mAb treatment (Fig. 5B), indicating a switch to more fatty acid oxidative metabolism during the weight loss observed during the acute treatment period (Fig. 5C). The same findings were observed in male DIO mice after acute R1c mAb treatment and when examined on day 13–15 after treatment start, no significant changes were observed on energy expenditure, RER or spontaneous locomotor activity (data not shown). R1c mAb treatment only modestly increased brown adipose tissue (BAT) peroxisome proliferator-activated receptor γ coactivator-1 (*Pgc-1*)*α* and uncoupling protein 1 (*Ucp1*) (compared to control mAb treated mice fed *ad libitum*) and *Pgc-1β* (compared to pair-fed control mAb) mRNA levels (Fig. 5D). R1c mAb also elevated WAT *Pgc-1β* mRNA levels but the WAT *Ucp1* mRNA levels were below detection limits. Collectively, these data show that the R1c mAb mediated body weight loss is primarily driven via a reduction in food intake and not enhanced energy expenditure.

### Role of hypothalamic neuronal circuits for the effects of R1c mAb

**Figure 6:**
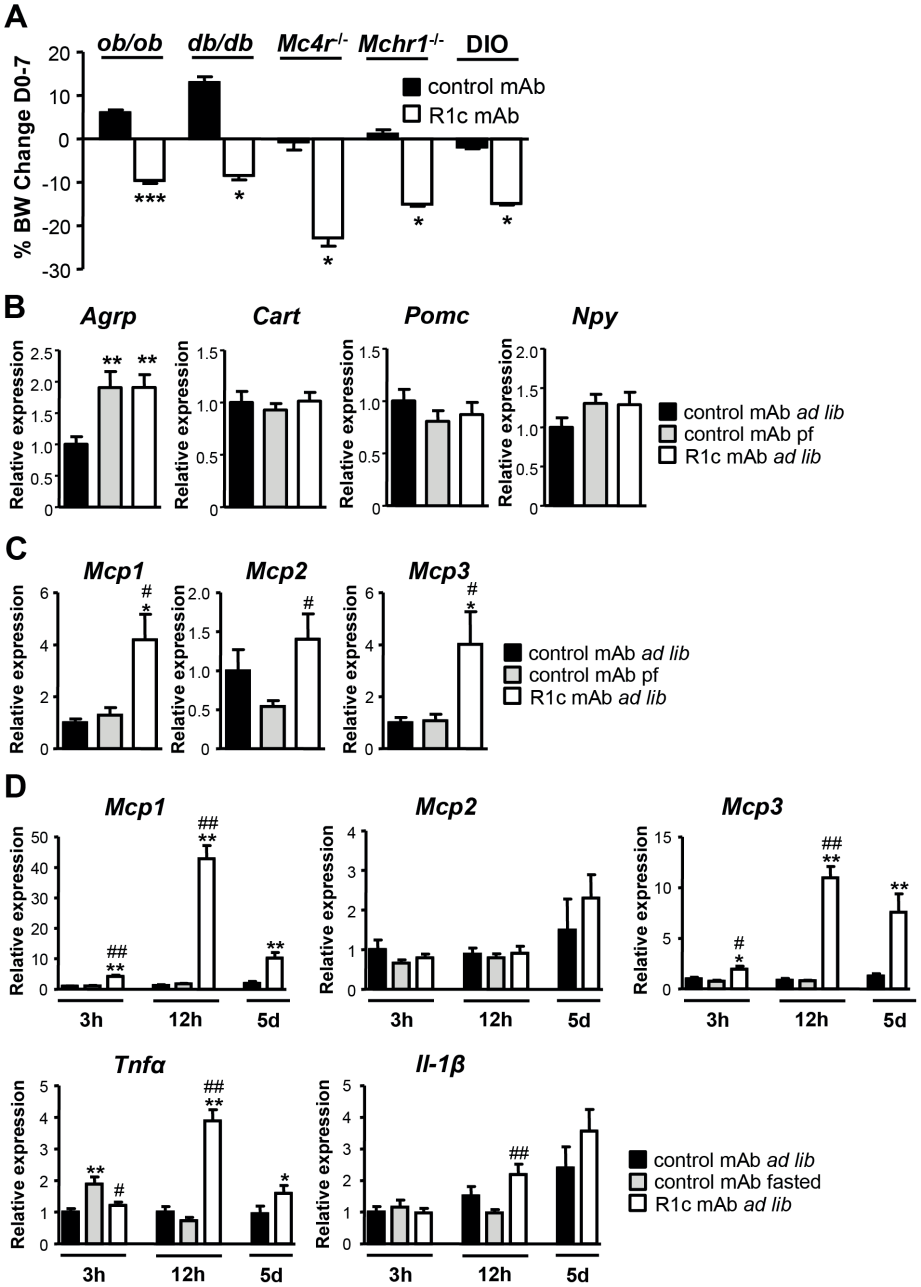
FGFR mAb effects in genetically obese mice and hypothalamic expression of neuropeptides and cytokines. (A) Change in body weight one week after a single injection of R1c mAb or control mAb (*n* = 8 for *ob/ob*, *n* = 4 for others, 10 mg/kg s.c.) in mice lacking leptin (*ob/ob*), functional leptin receptor (*db/db*), melanocortin 4 receptor (*Mc4r* ^-/-^) or melanin-concentrating hormone receptor 1 (*Mchr1* ^-/-^), compared to DIO mice. *P<0.05 and ***P<0.001 *vs*. control mAb, Mann Whitney *U*-test. (B) Neuropeptide mRNA expression and (C) Cytokine mRNA expression in hypothalamus, 4 d after a single injection of R1c mAb in female DIO mice fed *ad libitum* (*ad lib*) or control mAb treated female DIO mice fed *ad lib* or pair-fed (pf) twice daily to match the food intake in R1c mAb treated female DIO mice fed *ad lib* (*n* = 6, 3 mg/kg s.c.). *P<0.05, **P<0.01 *vs*. control mAb *ad lib* group; ^#^ *P*<0.05 *vs*. control mAb pf group, Mann Whitney *U*-test. (D) Cytokine mRNA expression in hypothalamus 3 h, 12 h and 5 d after a single injection of R1c mAb in female DIO mice fed *ad lib* compared to control mAb treated mice fed *ad lib* or fasted for the duration after the treatment (*n* = 6, 10 mg/kg i.v.). *P<0.05 and **P<0.01 *vs*. control mAb *ad lib* group; ^#^ *P*<0.05 and ^##^ *P*<0.01 *vs*. control mAb *fasted* group, Mann Whitney *U*-test. Values are means ± SEM.

R1c mAb also decreased the body weight in leptin-deficient *ob/ob* mice, leptin receptor-mutant *db/db* mice, melanocortin 4 receptor (*Mc4r*)-deficient mice and melanin-concentrating hormone receptor 1 (*Mchr1*)-deficient mice (Fig. 6A). In addition, R1c mAb did not affect hypothalamic mRNA expression levels of cocaine- and amphetamine-regulated transcript (*Cart*), pro-opiomelanocortin (*Pomc*), neuropeptide Y (*Npy*), corticotropin-releasing hormone (*Crh*), *Mch*, or Orexin (*Hcrtr*) in DIO mice compared with control mAb treated DIO mice fed either *ad libitum* or pair-fed to match the food intake of R1c mAb treated DIO mice (Fig. 6B and data not shown). Hypothalamic mRNA expression of agouti-related protein (*Agrp*), increased in both R1c mAb and pair-fed control mAb treated DIO mice compared to control mAb treated DIO mice fed *ad libitum* (Fig. 6B). These results indicate that R1c mAb might cause food intake inhibition via other mechanisms than the classical hypothalamic neuronal circuits known to be of importance for regulation of food intake.

### Effects of R1c mAb on hypothalamic cytokine levels

In conditions like cachexia, cytokines are strongly implicated in the mechanism controlling the cessation of food intake, reviewed in [11]. Interestingly, R1c mAb markedly elevated Monocyte chemoattractant protein (*Mcp*)1, *Mcp2* (*Ccl7*), *Mcp3* (*Ccl8*) and C-X-C motif chemokine 5 (*Cxcl5*) mRNA levels in the hypothalamus 4 d post-dosing while the mRNA expression levels of tumor necrosis factor α (*Tnfα*), interleukin (*Il*)-1β, *Il-6*, C-C chemokine receptor (*Ccr*)2, *Ccr5*, *Cd11c*, stromal-cell derived factor 1 (*Cxcl12*), *Cx3cl1*, interferon γ (*Ifnγ*), leukemia inhibitory factor (*Lif*), prostaglandin-endoperoxide synthase (*Ptgs*, cyclooxygenase)1, *Ptgs2*, suppressor of cytokine signaling 3 (*Socs3*), or toll-like receptor 4 (*Tlr4*) were unaltered compared to pair-fed control mAb treated DIO mice (Fig. 6 and data not shown). Thus, R1c mAb treatment induced expression of a specific subset of chemokines in the hypothalamus.

Detailed studies showed that *Mcp1* and *Mcp3* mRNA levels were elevated in the hypothalamus already 3 h after R1c mAb i.v. injection, coinciding with initiation of food intake suppression. The *Mcp1* and *Mcp3* expression levels were highly elevated 12 h post-injection and remained increased 5 d post-injection while the related cytokine *Mcp2* was not increased by R1c mAb at any time point (Fig. 6D). Furthermore, *Tnfα* mRNA levels were elevated in the hypothalamus 12 h and 5 d post-injection, whereas *Il-1β* was only significantly increased 12 h after R1c mAb injection compared with fasted control mAb treated DIO mice. Importantly, fasting in itself did not affect hypothalamic mRNA expression levels of *Mcp1*, *Mcp2*, *Mcp3*, or *Il-1β* (Fig. 6D). Liver *Mcp1* mRNA expression and plasma MCP1 levels were not elevated by R1c mAb treatment at any time point (data not shown). Thus, R1c mAb induced a rapid and marked elevation in hypothalamic *Mcp1* and *Mcp3* cytokine expression in a time frame that coincides with initiation of food intake suppression.

### R1c mAb accumulation and activation of neurons and kinases in the brain

**Figure 7:**
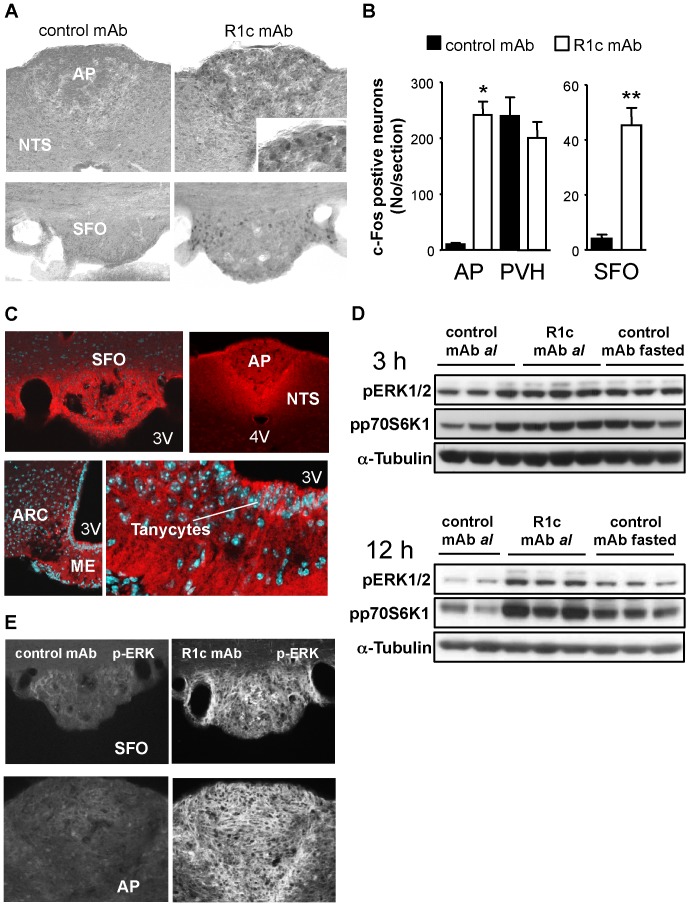
R1c mAb effects on c-Fos activation, human IgG accumulation and phosphorylation of ERK1/2 and p70S6K1 in the brain. (A) Representative photomicrographs and (B) Quantification of R1c mAb induced neuronal activity as measured by c-Fos labelling 3 h after a single injection of R1c mAb or control mAb in female DIO mice (*n* = 3–5, 10 mg/kg i.v., all mice fasted during the 3 h treatment period). *P<0.05, **P<0.01 *vs*. control mAb, Mann Whitney *U*-test. Values are means ± SEM. (C) Accumulation of R1 mAb as shown by representative confocal microscope images detecting human IgG 7 h after a single injection of R1c mAb in female DIO mice (10 mg/kg i.p.). (D) Western blot analyses of phosphorylation of ERK1/2 and p70S6K1 in the hypothalamus 3 h and 12 h post-injection with R1c mAb in female DIO mice fed *ad libitum* (*al*) compared to control mAb treated mice fed *al* or fasted for the duration after the injection. All mAbs were delivered at 10 mg/kg, i.v. and α-Tubulin is given as protein loading control. (E) Representative confocal microscope images showing phosphorylated ERK (p-ERK) 7 h after a single injection of R1c mAb or control mAb in female DIO mice (10 mg/kg i.p., all mice fasted during the 7 h treatment period). AP, area postrema; ARC, arcuate nucleus; ME, median eminence; NTS, nucleus tractus solitaries; PVH, paraventricular hypothalamic nucleus; SFO, subfornical organ.

To further explore which specific neuronal circuits and areas within the brain that were affected by R1c mAb treatment, we first measured the expression of the immediate early gene *c-Fos*, a reliable marker of neuronal activity. Interestingly, in the area postrema and subfornical organ, there was a strong R1c mAb induced neuronal c-Fos activation 3 h (Fig. 7A–B) and 12 h after an i.v. injection and 7 h after an i.p. injection (data now shown). In contrast, the c-Fos signal in the paraventricular nucleus of the hypothalamus was similar in both R1c and control mAb treated mice (Fig. 7B). We could further confirm accumulation of human IgG in the median eminence with a distinct labeling of tanycytes at the base of the 3^rd^ ventricle and adjacent arcuate nucleus, as well as in the subfornical organ and area postrema (Fig. 7C).

We hypothesized that if the dramatic increase in hypothalamic expression of certain cytokines upon R1c mAb treatment was physiologically important, we could expect to also see hypothalamic activation of ERK1/2 and p70S6K1 signaling pathways shown to be important for regulation of food intake [12]–[15]. R1c mAb increased hypothalamic phosphorylation of ERK1/2 and p70S6K1 12 h post-injection, with a trend also 3 h post-injection (Fig. 7D). The increased phosphorylation of ERK1/2 and p70S6K1 was not apparent 30 min post-injection when food intake was not affected (data not shown). Thus, activation of protein kinases ERK1/2 and p70S6K1 was associated with the initial time-course of food intake suppression caused by R1c mAb treatment. In addition, using double-labeling immunohistochemistry for p-ERK1/2 and c-Fos, R1c mAb treatment was found to increase the phosphorylation of ERK1/2 in each of the three circumventricular organs, particularly in the area postrema and subfornical organ compared to control mAb treated mice (Fig. 7E). Immunoreactivity seemed to be mainly concentrated in dendritic/axonal processes rather than in neuronal cytoplasm. Thus, increased phosphorylation of ERK1/2 implicates the ERK1/2 MAP kinase pathway within neurons of the circumventricular organs and in the mediobasal hypothalamus in the food intake suppressing effect of R1c mAb treatment.

**Figure 8:**
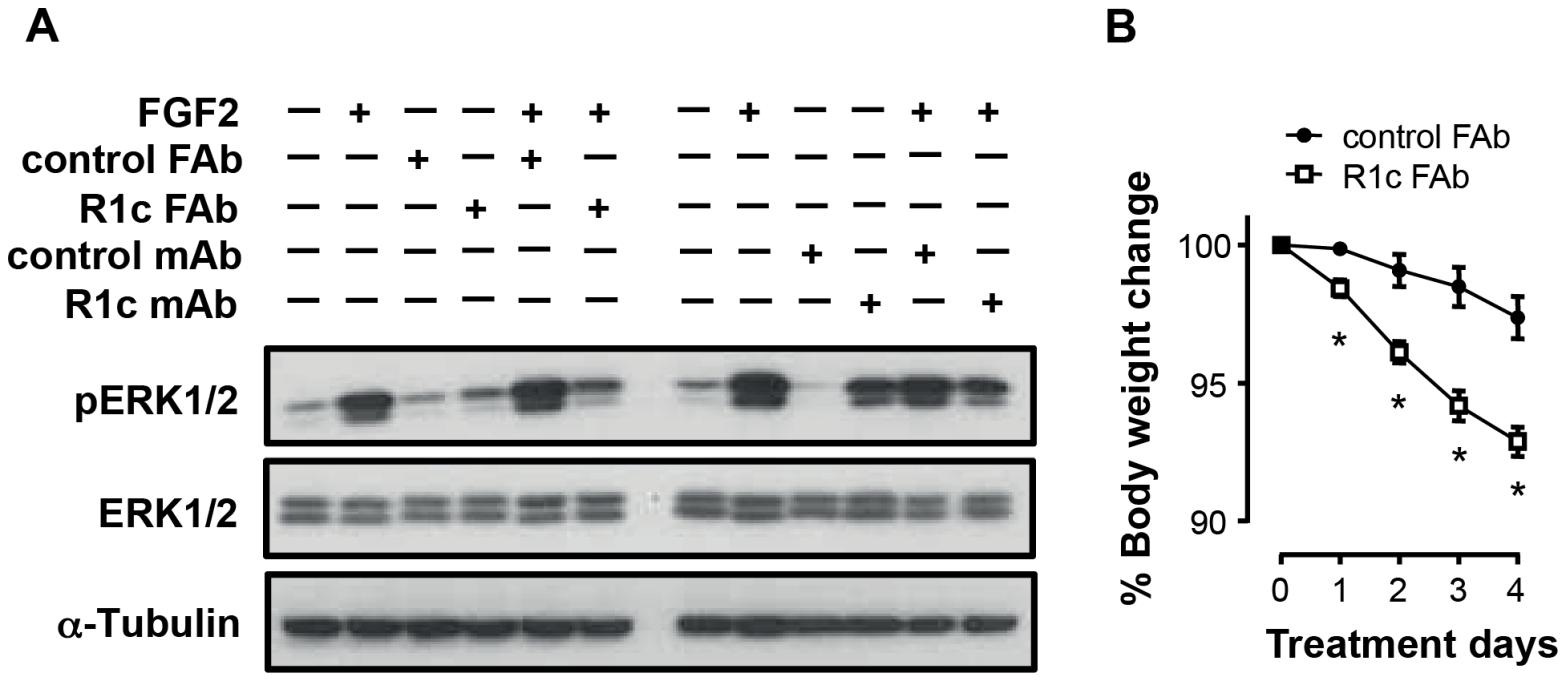
FGFR1c FAb fragment effects on FGFR1 down-stream signalling and body weight. (A) Representative Western blot analyses on FGFR1c (R1c) Fab, control FAb, R1c mAb, control mAb (50 µg/ml) and FGF2 (10 ng/ml) treated undifferentiated 3T3-L1 cells. (B) Body weight following repeated injection of either R1c FAb or control FAb fragments in female DIO mice (160 mg/kg/day, sc, BID, *n* = 4). *P<0.05, R1c FAb *vs*. control FAb at corresponding treatment day, Mann Whitney *U*-test. Values are means ± SEM.

R1c mAb agonism could be due to induced FGFR1c homodimerization leading to ERK1/2 phosphorylation similar to endogenous agonists [16]. In an attempt to see if FGFR1c antagonism only could cause weight-loss, a FAb fragment of the R1c mAb was generated. In contrast to R1c mAb, R1c FAb did not induce phosphorylation of ERK1/2 in 3T3-L1 cells but still antagonized FGF2 induced ERK1/2 phosphorylation (Fig. 8A). In addition, R1c FAb still caused body weight loss in DIO mice (Fig. 8B), showing that FGFR1c antagonism alone can cause body weight loss.

## Discussion

Previously described FGFR1c monoclonal antibodies affecting body weight in animals have been claimed to be either purely antagonistic (IMC-A1) [4] or agonistic [5]. Here we describe that an anti-FGFR1c monoclonal antibody can possess both antagonistic and agonistic properties. R1c mAb was found to selectively bind FGFR1c and to prevent binding of FGF1, FGF2, FGF4, FGF5, and FGF6 to FGFR1c and to inhibit FGF2, FGF19, and FGF21 induced proliferation. R1c mAb also antagonized FGF2 induced phosphorylation of FGFR and multiple downstream signaling molecules in both neuronal N46 cells and 3T3-L1 cells. Unexpectedly, R1c mAb also increased phosphorylation of ERK1/2 in both undifferentiated and differentiated 3T3-L1 cells and in WAT after *in vivo* treatment similar to the agonistic FGFR1 mAb described by Wu *et al* [5]. Recently, another “FGF21 mimetic” mAb targeting β-Klotho was found to decrease body weight and improve glucose homeostasis in cynomolgus monkeys [17]. At present, it is a bit unclear how R1c mAb can have dual antagonistic/agonist actions but it does not seem to be related to absence or presence of β-Klotho since β-Klotho was present in differentiated 3T3-L1 cells but not in undifferentiated 3T3-L1 cells (data not shown). However, R1c FAb did not induce ERK1/2 phosphorylation in 3T3-L1 cells, indicating that R1c mAb agonistic action is due to induced FGFR1c homodimerization.

R1c mAb treatment modestly increased *Pgc-1α*, *Pgc-1β* and *Ucp1* mRNA levels in BAT but did not affect total energy expenditure in contrast to the agonistic FGFR1 mAb described by Wu *et al* [5]. R1c mAb had however marked effects on food intake and FGFR1 is expressed in many areas in the brain including areas where R1c mAb accumulated such as the circumventricular organs, and in the hypothalamus [18] and *Fgfr1c* has been shown to be the dominant isoform of *Fgfr1* in the hypothalamus [4]. R1c mAb treatment caused potent body weight loss in DIO mice and the weight loss could be accounted for entirely by decreased food intake in a pair-feeding experiment. We further showed that R1c mAb also caused marked body weight loss in *ob/ob* and *db/db* mice and in mice lacking the *Mc4r* or *Mchr1*. In addition, R1c mAb did not affect hypothalamic expression levels of *Cart*, *Npy*, *Crh*, *Mch*, *Hcrtr/Orexin*, or *Agrp* indicating that R1c mAb could cause food intake inhibition via other mechanisms than through classical hypothalamic neuronal circuits known to be of importance for regulation of food intake [19].

Although we cannot exclude that circulating immune cells could play a role in the R1c mAb mediated increase in hypothalamic expression levels of *Mcp1*, *Mcp3*, *Tnfα*, and *Il-1β*, circulating levels of MCP1 and liver expression of *Mcp1* were unaffected, indicating a centrally mediated effect. CNS inflammation has paradoxically been found to promote both positive and negative energy balance [20]. HFD feeding promoted low grade hypothalamic inflammation in experimental animals [21]–[26] while pharmacological or genetic inhibition of JNK [21], IKKβ/NF-κB [24], [25], toll-like receptor 4 (TLR4) [23], or myeloid differentiation factor 88 (MyD88) [22] decreased food intake leading to body weight loss. Furthermore, short-term HFD feeding has been found to cause hypothalamic reactive gliosis and neuronal injury in mice and hypothalamic gliosis was also observed in obese humans [26]. On the other hand, conditions associated with cachexia such as certain types of cancer, anorexia, fever, anhedonia or exposure to bacterial endotoxins have also been associated with increased levels of pro-inflammatory cytokines [11]. Lipopolysaccharide (LPS) - or muramyl dipeptide-induced anorexia increased hypothalamic gene expression of several cytokines [27] and central administration of MCP1, RANTES, IL-8, or IL-1β has been shown to decrease food intake [28], [29]. Both MCP1 and its receptor CCR2 are expressed in astrocytes and neurons in the hypothalamus and peripheral LPS administration was found to increase MCP1 binding in the hypothalamus [30], [31]. R1c mAb treatment did not affect core body temperature, energy expenditure, spontaneous locomotor activity, muscle wasting, general behavior or systemic MCP1 levels arguing against general inflammation-induced cachexia as a mechanism for R1c mAb mediated inhibition of food intake and body weight loss. Rather, the R1c mAb mediated marked induction of *Mcp1* and *Mcp3* in the hypothalamus coinciding with the initiation of food intake suppression could have direct effects on food intake inhibition. Interestingly, Shirazi *et al* recently showed that glucagon-like peptide 1 (GLP-1, exendin-4) mediated suppression of food intake and body weight was mediated by central elevation of IL-6 and IL-1 [32], indicating that specific hypothalamic cytokines could play a broader role in controlling food intake and body weight in response to different pharmacotherapies than previously appreciated.

FGF1 and FGF2 have been implicated in the central regulation of food intake. FGF1 levels were markedly increased in the cerebrospinal fluid following a meal or i.p. injection of glucose and i.c.v. administration of FGF1 or FGF2 decreased food intake in rats [33]–[35]. Administration of polyclonal anti-FGFR1 antibodies into the bilateral lateral hypothalamic area increased food intake in rats [36], indicating that the R1c mAb mediated decrease in food intake could at least partly be due to an FGFR1c agonistic action. Alternatively, the effect of R1c mAb on food intake inhibition may be due to an FGFR1c antagonistic action as also demonstrated using an antagonistic R1c FAb fragment. FGF1 and FGF2 are abundant in the brain [18] and additional administration via i.c.v. injections of FGF1 or FGF2 [33]–[35] may result in high ligand concentrations in the brain. Excessive ligand concentrations may act in an antagonistic mode and other FGFRs than FGFR1c may be important for the FGF signalling [37].

Clearly, effects of R1c mAb on the area postrema could be responsible for its food intake and body weight-reducing effects, as this circumventricular area in the caudal brainstem is intimately related to both ascending and descending medullary structures involved in the control of food intake and energy balance regulation, such as the nucleus of the solitary tract and the hypothalamus. The strong activation of subfornical organ neurons was unexpected, as the role of this circumventricular structure in food intake and energy balance regulation is less well known. However, more recently a case has been made for such a role on the basis of strong effects of relevant peptides such as leptin and orexin on neurons in the subfornical organ [38]–[40]. It would be certainly worthwhile to pursue the role of this area in the weight-reducing effects of the R1c mAb in addition to the activation of tanycytes lining the ventral portions of the 3^rd^ ventricle with communication to the basomedial hypothalamus. Interestingly in this respect, FGF2 was recently found to enhance proliferation of hypothalamic 3^rd^ ventricle α-tanycytes *in vivo* [41]. Additionally, Balland *et al* recently showed that re-establishing ERK signalling in median eminence tanycytes (which is decreased in DIO), restored both leptin transport to the hypothalamus and leptin sensitivity [42]. It would thus be interesting for future studies to see if R1c mAb treatment could affect leptin transport to the hypothalamus and leptin sensitivity in the obese state.

R1c mAb treatment activated the FGFR1c downstream signalling proteins ERK1/2 and p70S6K1 via phosphorylation in the hypothalamus associated with the initial time-course of hypothalamic cytokine induction and suppression of food intake and we could also detect phosphorylation of ERK1/2 in the circumventricular organs. Leptin has also been found to activate ERK in the hypothalamus and pharmacological blockade of ERK reversed the anorectic and weight-reducing effects of leptin [14]. Similarly, amylin and cholecystokinin (CCK) have been found to activate ERK in area postrema and nucleus tractus solitarius, respectively and ERK inhibition attenuated the ability of amylin and CCK to reduce food intake [13], [15]. Overexpression of constitutively active p70S6K1 in mediobasal hypothalamus has been shown to decrease food intake protecting against HFD-induced obesity, fat deposition and insulin resistance [12]. Thus, R1c mAb mediated suppression of food intake could be due to activation of ERK1/2 and p70S6K1 in the hypothalamus.

R1c treatment markedly improved glucose tolerance in DIO mice. This was most likely due to the decrease in food intake and body weight loss since pair-fed control mAb treated DIO mice displayed similar improvements in fasting plasma glucose and insulin levels as R1c mAb treated DIO mice. Furthermore, R1c mAb treatment did not influence pancreatic β and α cell mass or islets morphology. Pancreatic overexpression of dominant-negative FGFR1c using the *Ipf1*/*Pdx1* promoter has previously been shown to decrease the number of β-cells, change islets morphology, and to cause diabetes in mice [43]. Marked overexpression of dominant-negative FGFR1c already in the developing pancreas may have caused developmental pancreas derangements as compared to treatment of DIO mice with R1c mAb.

In summary, we have generated a novel human monoclonal antibody directed against FGFR1c, R1c mAb, having dual antagonistic/agonistic properties. Treatment of DIO mice with R1c mAb ameliorated obesity and glucose intolerance due to decreased food intake and body weight loss. The decreased food intake was associated with increased hypothalamic *Mcp1* and *Mcp3* expression and phosphorylation of ERK1/2 and p70S6K1.
